# Assessing willingness to pay for health care quality improvements

**DOI:** 10.1186/s12913-015-0678-6

**Published:** 2015-02-01

**Authors:** Md Sadik Pavel, Sayan Chakrabarty, Jeff Gow

**Affiliations:** Department of Economics, Shahjalal University of Science & Technology, Sylhet, 3114 Bangladesh; Australian Digital Futures Institute (ADFI), University of Southern Queensland, Toowoomba, QLD 4350 Australia; Australian Centre for Sustainable Business and Development, University of Southern Queensland, Springfield, QLD 4300 Australia; School of Commerce, University of Southern Queensland, Toowoomba, QLD 4350 Australia; Research Associate, Health Economics and HIV/AIDS Research Division (HEARD), University of KwaZulu-Natal, Westville Campus, Durban, South Africa

**Keywords:** Contingent valuation (CV), Willingness to pay (WTP), Health care, Quality attributes, Bangladesh, Classification code, I11, I18

## Abstract

**Background:**

Contingent valuation (CV) is used to estimate the willingness to pay (WTP) of consumers for specific attributes to improve the quality of health care they received in three hospitals in Bangladesh.

**Methods:**

Random sample of 252 patients were interviewed to measure their willingness to pay for seven specified improvements in the quality of delivered medical care. Partial tobit regression and corresponding marginal effects analysis were used to analyze the data and obtain WTP estimates.

**Results:**

Patients are willing to pay more if their satisfaction with three attributes of care are increased. These are: a closer doctor-patient relationship, increased drug availability and increased chances of recovery. The doctor patient relationship is considered most important by patients and exhibited the highest willingness to pay.

**Conclusions:**

This study provides important information to policy makers about the monetary valuation of patients for improvements in certain attributes of health care in Bangladesh.

**Electronic supplementary material:**

The online version of this article (doi:10.1186/s12913-015-0678-6) contains supplementary material, which is available to authorized users.

## Background

The quality of health care, as well as people’s preferences for health care, has changed in Bangladesh over the past 20 years. Since the 1990s’, private sector health care facilities have experienced rapid growth and currently the private for-profit sector accounts for 80% of the more than 3500 hospitals in Bangladesh and this rate is continuing to increase. More than 100 new private clinics and hospitals and 200 new diagnostic centres open every year [1]. At present there are 53 government and private medical colleges in Bangladesh [1], most of them situated in large tertiary hospitals in divisional cities. However, given widespread poverty, only 52% of ill people visit hospital annually and only 21% of ill people visited hospital more than three times in a year [2]. There are two main reasons for people not attending hospital when ill: i) the high user fees for private sector health care and ii) the failure to receive appropriate health care in government hospitals due to overcrowding and lack of resources. This increasing reliance on private provision of health care in a nation with an annual per capita income just above $USD1000 means that many miss out on hospital care regardless of need, because of their poverty.

Given this situation a contingent valuation (CV) study was designed to assess peoples’ willingness to pay (WTP) for health care quality improvements. This is a demand based approach to describe consumer preferences by observing their potential purchasing behavior. The results of this study will be useful for Government and private sector providers in allocating their funds in health care and setting appropriate user fees. This will also provide some complementary information for health care providers to develop co-payment schedules and improve health care facilities along the lines that consumers’ desire.

To enable this to occur a CV questionnaire was designed to assess the value of improving quality of hospital’s health care from the patients’ perspective. Contingent valuation is the most commonly used stated preference technique to assess patients’ preferences [3] through eliciting their WTP. A set of quality attributes was used to specify the nature and degree of quality improvements that are valued by patients. The demand for healthcare can be better assessed by evaluating consumers’ willingness to pay.

## Methods

### Contingent valuation method

Facing the increasing cost of health services and rising demand for health services, policy makers are interested in measuring the passive use value of health care services. The economic value that arises from a change in the quality of services which is not reflected in observational behavior [4] is often not captured. The CV method is the most widely used method to measure passive use value [5].

CV can measure the value that consumers place on certain aspects or attributes of health care services [6]. CV is often referred to as a stated preference model [7,8] in contrast to price-based revealed preference model [9]. The CV model is utility based and people are asked how much money they would be willing to pay to maintain or improve services or activities.

The CV method is a survey-based, hypothetical and direct method to elicit monetary value for improvements in goods or services [10]. CV questions are used to estimate the demand function or the willingness to pay distribution of consumers [5].

A CV questionnaire was designed to assess consumers’ valuation of improving the quality of hospital services. Improvements over seven quality attributes were separately assessed using a decomposed valuation scenario [11], the attributes and their corresponding measurement scales with hypothesis are downloadable as an Additional file 1. An implicit assumption of the decomposed valuation method is that utility variations following improvements in one attribute do not depend on the levels of other quality attributes [12,13]. WTP questions were asked in two stages: patients were first asked whether they would be willing to pay an extra user fee to benefit from a specific improvement, and only in case of a positive answer, were they then questioned about their maximum WTP; the WTP valuation process is downloadable as an Additional file 2. Finally, individual demographic and socioeconomic characteristics, including, gender, age, education (number of formal schooling years completed), marital status, location, employment status and household monthly income were collected.

### Study population and selection method

Data were collected in 2011 via face to face interviews in Sylhet, a major city in north-eastern Bangladesh. As a divisional city, people from surrounding areas also received health care in Sylhet. This city was chosen for data collection as it has medical training colleges and public hospitals and many private clinics. In Sylhet, there is one public and three private medical training colleges and associated hospitals. Patients were randomly selected amongst patients seeking care in three: MAG Osmani Medical College Hospital, Jalalabad Ragib-Rabeya Medical College & Hospital and Women’s Medical College & Hospital.

The sample consists of 252 patients from three medical college hospitals. Sampling design for this study followed a previously successful methodology [8]: patients were randomly selected and interviewed immediately after their consultation. Ten enumerators (university students) were trained to collect data. A serial number was assigned to each patient before their consultation and patients were randomly chosen. Taking a random sample of patients did not lead to any sample selection bias and also any potential identification problem during the analysis was avoided. Enumerators waited outside the doctor’s office for the randomly assigned patient to exit. Any adult patient was eligible to take part in the interview. Verbal informed consent was obtained before proceeding with the interview. When the patient was a child, the accompanying adult person answered the questionnaire. Enumerators provided some basic information to patients about the research study to get their cooperation. No inducement, financial or otherwise, was offered. The ethics committee of the Medical Faculty, Shahjalal University of Science & Technology, approved the study.

### Analysis

#### Tobit regression analysis

Tobit regression analysis assumes that the dependent variable has a number of its values clustered at a limiting value, usually zero. The Tobit model has the advantage of being able to efficiently estimate the relationship between an explanatory variable and some (censored) dependent variable to estimate the probability of a dependent variable being at or below (above) a limit [14,15].

Tobit regression analysis for limited dependent variables [16] examined the association between stated WTP values and patients’ demographic, socioeconomic characteristics. This is preferred to the ordinary least square (OLS) estimator which fails to account for qualitative differences between the limit observations (those with zero WTP) and the non-limit observations (those with WTP > 0), leading to erroneous estimation of the marginal effects [17]. When WTP questions are “open ended” and the nature of the dependent variables are “continuous with censoring at zero”, the most appropriate estimation technique is limited dependent variable with Tobit model [18]. The independent variables in the model are listed in Table 1. Seven different Tobit regressions were conducted; each of the regressions was followed by a RESET test [19].

**Table 1:** **Attributes (independent variables) specification and levels**

GPVFAR	Geographical proximity; 1 for “Very Far”, 0 for otherwise
GPFAR	Geographical proximity; 1 for “Far”, 0 for otherwise
GPAVG	Geographical proximity; 1 for “Average”, 0 for otherwise^a^
WTVLONG	Waiting time; 1 for “Very long”, 0 for otherwise
WTLONG	Waiting time; 1 for “Long”, 0 for otherwise
WTAVG	Waiting time; 1 for “Average”, 0 for otherwise
WTNLONG	Waiting time; 1 for “Not long”, 0 for otherwise^b^
ATTDVBAD	Attitude of hospital staff; 1 for “Very bad”, 0 for otherwise
ATTDBAD	Attitude of hospital staff; 1 for “Bad”, 0 for otherwise
ATTDGOOD	Attitude of hospital staff; 1 for “Good”, 0 for otherwise^c^
SAMNEVER	Seeing the same doctor; 1 for “Never”, 0 for otherwise
SAMRARE	Seeing the same doctor; 1 for “Rarely”, 0 for otherwise
SAMEOFTN	Seeing the same doctor; 1 for “Often”, 0 for otherwise^d^
DPRSC	Doctor–patient relationship; average of five items’ scores multiplied by 20, range [20,100]
DRUGNONE	Drug availability; 1 for “None of them”, 0 for otherwise
DRUGSOME	Drug availability; 1 for “Some of them”, 0 for otherwise^e^
RECOVSC	Chance of recovery; average of five items’ scores multiplied by 20, range [20,100]
SEX	Sex; 1 for female, 0 for male
AGE	Age; in years
EDUC	Education; number of schooling years
INCOME	Income in Bangladeshi Taka (BDT) (continuous)
LOCATION	Location; 1 for rural, 0 for urban
NATURE	Nature; 1 for private, 0 for government
REASON	Reason of medical visit; 1 for acute reason, 0 for otherwise

Notes: ^a^Geographical proximity = “Close” and “Very close” are combined and included in the constant.

^b^Waiting time = “Not long at all” is included in the constant.

^c^Attitude = “Excellent” is included in the constant.

^d^Seeing the same doctor = “Always” is included in the constant.

^e^Drug Availability = “All” is included in the constant”.

Firstly, seven partial Tobit regressions corresponding to seven different attributes were analyzed to estimate the “beta” coefficients which explain expected willingness to pay (WTP) for each attribute and to show how WTP varies with socio-economic characteristics. Secondly, the marginal effects β′ and β″ were estimated where, β′ explained the marginal effects for the probability of being uncensored and β″ explained the marginal effects for the expected WTP value conditional on being uncensored: *E* (WTP | WTP > 0).

## Results and discussion

### Descriptive statistics

Table 2 presents patients’ current estimation for the seven attributes used to measure the quality of services. Thirty percent of patients came to the hospital from a “very far” distance. The mean travel time to the hospital was about 65 minutes with significant variations between patients (±56 minutes). Patients declared that a travel time of about 23 minutes would be considered as “very close”. On average, patients waited 73 minutes (max = 240 minutes) before seeing the doctor. This was perceived as “long” or “very long” by 61% of total patients. Patients declared that a waiting time of less than 25 minutes would be perceived as “not long at all”. In general, 22.2% of patients felt that their treatment was “excellent” by the staff of the hospital. A lower portion of patients (10.7%) felt that they had received “bad” or “very bad” treatment.

**Table 2:** **Estimates of attributes characteristics**

**Attributes**	**Categories**	**N (%)**	**Mean (±S.D.)**
Geographical proximity	Very Far	76 (30.2%)	Time taken to reach hospital: 64.91 minutes (±56.82)
	Far	38 (15.1%)	
	Average	43 (17.1%)	
	Close or Very Close	95 (37.7%)	Travel time considered hospital located “Very Close”: 21.90 minutes (±14.48)
Waiting time	Very Long	87 (34.5%)	Waiting time before meet the doctor: 72.51 minutes (±59.89)
	Long	67 (26.6%)	
	Average	22 (8.7%)	
	Not Long	46 (18.3%)	
	Not Long at All	30 (11.9%)	Waiting time considered “Not Long at all”: 23.13 minutes (±14.47)
Attitude of hospital staff	Excellent	56 (22.2%)	Not Applicable
	Good	169 (67.1%)	
	Bad	22 (8.7%)	
	Very Bad	5 (2.0%)	
Seeing the same doctor	Always	57 (22.6%)	Not Applicable
	Often	73 (29.0%)	
	Rarely	14 (5.6%)	
	Never	17 (6.7%)	
	First visit	91 (36.1%)	
Doctor-patient relationship score	Not Applicable	Not Applicable	72.63 minutes (±14.16)
Drug availability	All	26 (10.3%)	Not Applicable
	Some	66 (26.2%)	
	None	160 (63.5%)	
Chance of recovery score	Not Applicable	Not Applicable	71.22 minutes (±11.48)

Table 3 represents patients’ assessment of the quality attribute “geographical proximity” using that attribute’s five categorical scales such as “hospital was very far from home” and another four measures.

**Table 3:** **Patient’s assessment of geographical proximity**

**Geographical proximity**	**Number of patients (N)**	**Mean (standard deviation) in minutes**
Hospital was “very far from home”	76	122.83 (51.46)
Hospital was “far from home”	38	84.34 (51.37)
Hospital was “at average distance from home”	43	43.72 (20.87)
Hospital was “close to home”	64	24.17 (15.43)
Hospital was “very close to home”	31	12.58 (6.09)

Table 4 represents patients’ assessment of the quality attribute “waiting time” in order that this attribute’s five categorical scales such as “very long”, “long” can be measured.

**Table 4:** **Patient’s assessment of waiting time**

**Waiting time**	**Number of patients (N)**	**Mean (standard deviation) in minutes**
Waiting time as “very long”	87	127.82 (55.91)
Waiting time as “long”	67	63.13 (42.11)
Waiting time as “average”	22	36.82 (27.45)
Waiting time as “not long”	46	33.30 (27.14)
Waiting time as “not long at all”	30	19.33 (18.41)

Only one-fourth of the patients (22.6%) were always examined by the same doctor; about 6% of the patients rarely meet, and about 7% have never met the same doctor in the hospital. Only 10.3% of patients were able to find all their medicine(s) within in the range of the registration fee or user fee; 26.2% found some and 63.5% did not find any of their medicine. Measuring the quality of DPR resulted in a mean score of 72.63 (±14.16), range [20,100]. Patients’ estimation of a mean chance of recovery was 71.22 (±11.48), range [20,100].

Examining the WTP for each of the seven attributes in Table 5 showed that the highest willingness to pay for improvements was for “drug availability” at 123.69 BDT. The lowest stated WTP values (21.39 BDT) concerned proposed improvements to staff attitudes. Patients were willing to pay most for the three quality attributes “DPR”, “drug availability” and RECVSC. All “zero” values given by patients were included in the analysis.

**Table 5:** **Patients willingness to pay (WTP) for improvements in each of the attributes and mean WTP values per attribute**

**Attribute**	**Positive WTP (>0) N (%)**	**WTP (BDT): Mean (±S.D.)** ^**a**^
Geographical proximity	168 (66.7%)	29.25 (±38.63)
Waiting time	172 (68.3%)	26.65 (±34.93)
Attitude of hospital staff	155 (61.5%)	21.39 (±24.16)
Seeing the same doctor	163 (64.7%)	26.05 (±36.02)
Doctor–patient relationship	239 (94.8%)	91.51 (±109.33)
Drug availability	231 (91.7%)	123.69 (±137.63)
Chance of recovery	222 (88.1%)	95.28 (±132.00)

Notes: ^a^Non-contributors (WTP = 0) were included in the calculated means.

### Tobit regression analysis estimates

The results from the seven Tobit regressions as outlined in Table 6 suggest the existence of a strong and highly significant association between stated WTP values and improvements in the seven different quality attributes.

**Table 6:** **Factors influencing partial WTP values**

**Independent variables**	**B (BSE.)**						
	**Geographical proximity**	**Waiting time**	**Attitude of hospital staff**	**Seeing the same doctor**	**Doctor-patient relationship** ^**a**^	**Drug availability**	**Chance of recovery** ^**a**^
Constant	- 19.52 (14.48)	−8.66 (15.04)	−30.26*** (11.01)	−25.87* (14.27)	−182.36*** (44.29)	−61.09 (42.84)	−320.86*** (67.22)
GPVFAR	58.95*** (10.11)	-	-	-	-	-	-
GPFAR	36.11*** (10.54)	-	-	-	-	-	-
GPAVG	21.89** (9.98)	-	-	-	-	-	-
WTVLONG	-	40.56*** (11.17)	-	-	-	-	-
WTLONG	-	36.77*** (11.01)	-	-	-	-	-
WTAVG	-	24.02* (14.00)	-	-	-	-	-
WTNLONG	-	7.46 (11.86)	-	-	-	-	-
ATTDVBAD	-	-	9.36 (16.10)	-	-	-	-
ATTDBAD	-	-	−19.65** (9.11)	-	-	-	-
ATTDGOOD	-	-	−20.22*** (5.48)	-	-	-	-
SAMNEVER	-	-	-	26.96** (12.82)	-	-	-
SAMRARE	-	-	-	−35.59** (15.88)	-	-	-
SAMEOFTN	-	-	-	−6.23 (7.98)	-	-	-
DPRSC	-	-	-	-	−1.05** (0.47)	-	-
DRUGNONE	-	-	-	-	-	99.61*** (33.37)	-
DRUGSOME	-	-	-	-	-	32.68 (32.13)	-
RECOVSC	-	-	-	-	-	-	−3.20*** (0.83)
SEX	2.611 (6.58)	5.58 (6.00)	−0.24 (4.65)	−3.34 (6.46)	−14.34 (13.34)	22.10 (17.55)	−33.96* (17.69)
AGE	−0.48*** (0.17)	−0.27* (0.15)	−0.17 (0.11)	−0.37** (0.16)	−0.59* (0.34)	0.05 (0.45)	−0.26 (0.44)
EDUC	0.71 (0.72)	1.21* (0.68)	0.08 (0.54)	0.39 (0.71)	−1.54 (1.50)	−1.37 (2.00)	−0.43 (1.95)
INCOME	0.00001 (0.0002)	0.0002 (0.0002)	0.0002 (0.0001)	0.0008*** (0.0002)	0.002*** (0.0004)	0.002*** (0.0006)	0.002*** (0.0006)
LOCATION	−8.91 (8.21)	2.15 (6.39)	−3.57 (4.83)	2.09 (6.76)	−21.53 (14.11)	−27.63 (18.65)	−15.84 (18.31)
NATURE	−2.63 (7.04)	−16.24** (6.96)	−11.76** (5.11)	−15.91** (7.08)	30.008** (14.11)	−17.35 (24.01)	22.70 (19.84)
REASON	−7.44 (5.20)	0.61 (4.79)	3.52 (3.64)	−4.90 (5.14)	−11.48 (10.83)	−15.71 (14.32)	−9.36 (14.22)
Number of observations	252	252	252	252	252	252	252
Number of censored observed	84	80	97	89	13	21	30
Log likelihood	−948.97	−951.70	−838.53	−920.69	−1460.22	−1483.94	−1425.54
Probability > χ^2^	0.0000	0.0000	0.0005	0.0000	0.0000	0.0001	0.0000
RESET (probability > F)	0.3819	0.6382	0.1206	0.6700	0.0271	0.1147	0.0414

Notes: B = coefficient, B S.E. = standard error of the coefficient.

*P < 0.10; **P < 0.05; ***P < 0.01.

^a^DPR score and Chance of Recovery score; range [20,100].

Patients are willing to pay for geographical proximity as shown by the values “very far”, “far” and “average” distance respectively of 58.95, 36.11 and 21.89 BDT. The first two results were significant at a 1% level and the latter was significant at the 5% level. This result suggests that patients living “very far” or “far” from the hospital were willing to pay more than those living “average” distances.

Similarly, patients are willing to pay for shorter waiting times as shown by the values for “very long”, “long”, “average” and “not long” which were respectively 46.56, 36.77, 24.02 and 7.46 BDT. The first two results were significant at the 1% level and the “average” was significant at 10% level. Those benefiting the most from reducing waiting times before meeting the doctor to a minimum, that is patients currently waiting “very long”, “long” and “average” before meeting the doctor were willing to pay the highest user fee increments to benefit from a “not long” waiting time. Patients are willing to pay for improvement staff attitudes as shown by the “very bad”, “bad”, and “good” were respectively 9.36, −19.65 and −20.22 BDT. Staff attitude for “bad” was significant at 5% level and “good” was significant at 1% level. Negative WTP indicates that when patients received “very bad” behavior from staff they were willing to pay a higher user fee (WTP = 9.36) but when patients received better behavior from the staff she/he was willing to pay a lower user fee (negative value). This signifies that there is a significant demand for better staff attitudes.

Patients were also willing to pay in order to be “never”, “rare” and “often” able to meet the same doctor in the hospital respectively 26.96, −35.59 and −6.23 BDT. Seeing the same health professional for “never” and “rare” was significant at the 5% level. However, those who “never” meet the same doctor had a positive WTP value, and declared higher WTP values, in comparison with those who “rare” meet, or “often” meet, the same doctor in the hospital. It can be argued that, those who “often” meet the same doctor might have estimated that it was not worthwhile to pay more just to see her/him every time because next time they will most probably meet the same one. On the other hand, those who have “rare” do not feel the advantage of meeting the same doctor every time. On the other hand, those who have “never” met the same doctor in the hospital feel the advantage of meeting the same doctor every time most highly.

Patients were willing to pay −1.05 BDT for doctor-patient relationship (DPRSC) to get sufficient information from the doctor. This result was significant at the 5% level. Similarly patients were willing to pay −3.20 BDT for the chance of recovery (RECOVSC) and that was significant at the 1% level. These results also suggest that when the patient is less satisfied from their relationship with the doctor, as assessed by the calculated DPR-score, and RECOV-score they were willing to pay more to get ‘proper’ treatment and to spend longer time with the doctor.

Finally, patients were willing to pay for drugs to be available. “None” and “some” were respectively 99.61 and 32.68 BDT. Drug availability for “none” was significant at the 1% level. Patients who did not find any of their prescribed medications in the hospital were willing to pay more than those who found “some” or “all” of their medications. Females were willing to pay more than males to benefit from improvements over the geographical proximity (2.611), waiting time (5.58) and drug availability (22.10) attributes. On the other hand, females were willing to pay less than males to benefit from improvements over the staff attitude (−0.24), see the same health professional (−3.34), doctor-patient relationship (−14.34) and chance of recovery (−33.96) attributes. Differences were not significant for the other attributes except chance of recovery attribute (p < 0.10). In the local context, females usually have less control over household resources, which may explain their lower stated WTP values.

Similarly, elderly patients were willing to pay less than younger patients for the all attributes except the drug availability attribute. Other attribute improvements are: geographical proximity (−0.48; p < 0.01), waiting time (−0.27; p < 0.10), staff attitude (−0.27; p < 0.10), seeing the same health professional (−0.37; p < 0.05), improved doctor-patient relationship (−0.59; p < 0.10) and improved chance of recovery (−0.26).

Higher educated patients were willing to pay more than lower educated patients to benefit from improvements for geographical proximity (0.71), waiting time (1.21; p < 0.10), staff attitudes (0.08) and seeing the same health professional (0.39) attributes. On the other hand, higher educated patients were willing to pay less than lower educated person to benefit from improvements in the doctor-patient relationship (−1.54), drug availability (−1.37) and chance of recovery (−0.43) attributes. Higher income earners were willing to pay more than lower income earners to benefit from improvements over all seven quality attributes: geographical proximity (0.00001), waiting time (0.0002), staff attitude (0.0002), see the same health professional (0.0008; p < 0.01), doctor-patient relationship (0.002; p < 0.01), drug availability (0.002; p < 0.01) and chance of recovery (0.002; p < 0.01).

The geographical location of the patient’s home played a role in their stated WTP values. Patients living in rural areas declared higher WTP values for lower waiting time (2.15) and able to meet the same health professional (2.09). On the other hand, rural patients were willing to pay less than urban patients to benefit from improvements over all other attributes: geographical proximity (−8.91), staff attitude (−3.57), doctor-patient relationship (−21.53), drug availability (−27.63) and chance of recovery (−15.84).

In general, patients receiving health care from private hospitals were willing to pay more to improve the doctor patient relationship (30.008; p < 0.05) and chances of recovery attributes (22.70), and less for geographical proximity (−2.63), waiting time (−16.24; p < 0.05), staff attitude (−11.76; p < 0.05), see the same health professional (−15.91; p < 0.05), and drug availability (−17.35) attributes, compared to those attending governmental facilities. Finally, patients coming to the hospital for an acute or common illness were willing to pay less than those who come to the hospital due to other reasons: geographical proximity (−7.44), and see the same health professional (−4.90), doctor-patient relationship (−11.48), drug availability (−15.71) and chance of recovery (−9.36) attributes were evidenced. On the other hand, patients coming to the hospital for an acute or common illness were willing to pay more than those who come to the hospital due to improvements in waiting times (0.61), and staff attitude (3.52) attributes.

### Marginal effects estimates

The marginal effects are presented in Table 7 [20]. The degree of quality improvement was significantly associated with the stated WTP values. This is evidence as to the construct validity of the method. In marginal effects for geographical proximity, results suggest that the probability that a patient living “very far” from a hospital would be willing to pay in order to have a “very close” hospital, was 39% greater than that of a patient living “very close” or “close” to a hospital, and this result was significant at the 1% level. Moreover, patients living “far” or at an “average” distance from the hospital were willing to pay, respectively, 15% and 17% greater than that of a patient living “very close” or “close” to a hospital. These results are significant at the 1% level and at the 5% level respectively. Moreover, those living “very far” from the center were willing to pay 30.65 BDT more at every visit to have a “very close” hospital (significant at the 1% level). Patients living “far” or at an “average” distance from the center were willing to pay, respectively, 18.87 and 10.73 BDT more at every consultation to have a “very close” hospital, where the former was significant at the 1% level and the latter was significant at the 5% level.

**Table 7:** **Marginal effects of factors influencing WTP values**

**Independent ** **variable**	**Geographical proximity**		**Waiting time**		**Attitude of hospital staff**		**Seeing the same doctor**		**Doctor-patient relationship**		**Drug availability**		**Chance of recovery**	
	**β′**	**β″**	**β′**	**β″**	**β′**	**β″**	**β′**	**β″**	**β′**	**β″**	**β′**	**β″**	**β′**	**β″**
GPVFAR	0.39***	30.65***	-	-	-	-	-	-	-	-	-	-	-	-
GPFAR	0.24***	18.87***	-	-	-	-	-	-	-	-	-	-	-	-
GPAVG	0.16**	10.73**	-	-	-	-	-	-	-	-	-	-	-	-
WTVLONG	-	-	0.31***	19.96***	-	-	-	-	-	-	-	-	-	-
WTLONG	-	-	0.28***	18.60***	-	-	-	-	-	-	-	-	-	-
WTAVG	-	-	0.18**	12.32	-	-	-	-	-	-	-	-	-	-
WTNLONG	-	-	0.06	3.47	-	-	-	-	-	-	-	-	-	-
ATTDVBAD	-	-	-	-	0.09	4.54	-	-	-	-	-	-	-	-
ATTDBAD	-	-	-	-	−0.23**	−7.68**	-	-	-	-	-	-	-	-
ATTDGOOD	-	-	-	-	−0.21***	−9.66	-	-	-	-	-	-	-	-
SAMNEVER	-	-	-	-	-	-	0.19***	13.46*	-	-	-	-	-	-
SAMRARE	-	-	-	-	-	-	−0.30**	−12.62***	-	-	-	-	-	-
SAMEOFTN	-	-	-	-	-	-	−0.05	−2.64	-	-	-	-	-	-
DPRSC	-	-	-	-	-	-	-	-	−0.002**	−0.62**	-	-	-	-
DRUGNONE	-	-	-	-	-	-	-	-	-	-	0.21***	56.03***	-	-
DRUGSOME	-	-	-	-	-	-	-	-	-	-	0.06	19.84	-	-
RECOVSC	-	-	-	-	-	-	-	-	-	-	-	-	−0.007***	−1.67***
SEX	0.02	1.17	0.04	2.52	−0.002	−0.10	−0.02	−1.44	−0.03	−8.39	0.04	13.11	−0.08**	−17.64**
AGE	−0.003***	−0.21***	−0.002*	−0.12*	−0.002	−0.08	−0.003**	−0.16**	−0.001*	−0.34*	0.0001	0.03	−0.0006	−0.13
EDUC	0.005	0.31	0.01*	0.54	0.0009	0.03	0.003	0.16	−0.004	−0.90	−0.002	−0.81	−0.001	−0.22
INCOME	1.13e-06	0.00006	1.88e-06	0.00009	2.67e-06	0.0001	6.7e-06***	0.0003***	6.23e-06***	0.001***	4.09e-06***	0.001***	5.26e-06***	0.001***
LOCATION	−0.06	−4.04	0.01	0.96	−0.40	−1.61	0.01	0.90	−0.05	−12.78	−0.05	−16.47	−0.03	−8.34
NATURE	−0.02	−1.18	−0.13**	−7.24**	−0.13**	−5.23**	−0.13**	−6.80**	0.07**	17.77**	−0.03	−10.21	0.05	11.94
REASON	−0.05	−3.34	0.005	0.27	0.03	1.54	−0.04	−2.11	−0.03	−6.75	−0.03	−9.27	−0.02	−4.90

Notes: β′ is the marginal effects for the probability of being uncensored and β″ is the marginal effects for the expected WTP value conditional on being uncensored: *E* (WTP | WTP > 0). *P < 0.10; **P < 0.05; ***P < 0.01.

Similar results were obtained for the waiting time attributes. Indeed, patients waiting “very long”, “long”, “average” and “not long” before meeting the doctor were willing to pay 31%, 28%, 18% and 6% respectively greater than that of a patient waiting “not long at all”. The first two results were significant at the 1% level and the “average” was significant at the 5% level. Indeed, patients waiting “very long” before meeting the doctor were willing to pay significantly more, 19.96 BDT, to improve the attribute (significant at the 1% level). Patients waiting “long”, “average”, and “not long” before meeting the doctor were willing to pay more 18.60, 12.32 and 3.47 BDT respectively, to improve the attribute to “not long at all”. The result for “long” was significant at the 1% level.

In marginal effects for Staff Attitudes, patients feeling they were treated “very badly” were willing to pay more than that of a patients feeling they are treated “excellent” by the staff of the hospital. But patients feeling they are treated “badly” and “good” respectively by the staff of the hospital were willing to pay significantly less by 23% and 21% respectively, than that of patients feeling that they are treated “excellent” by staff. These results were significant at the 5% level and at the 1% level respectively. Moreover, the patients feeling they are treated “very badly” by staff, were willing to pay more (4.54 BDT) to improve this attribute. Patients feeling they were treated “badly” and “good” by the staff of the hospital expressed a negative willing to pay −7.68 BDT (significant at the 5% level) and −9.66 BDT respectively. That means WTP decreases if staff attitudes turned from “Very Bad” to “Excellent”.

Patients were also willing to pay in order to be “always” able to meet the same doctor in the hospital. However, those who “never” meet the same doctor had a higher probability of stating a positive WTP value, and declared higher WTP values that is 19% greater (significant at the 1% level), in comparison with those who “rare” (−30%) (significant at the 5% level) meet, or have “often” (−5%) meet, the same doctor in the hospital. Moreover, patients meeting the same doctor “never” were willing to pay (13.46 BDT) more at every visit (significant at the 10% level). Patients meeting the same doctor “Rare” and “Often” at the hospital expressed a negative willing to pay −12.62 BDT (significant at the 1% level) and −2.64 BDT respectively. It can be argued that, those who “often” might have estimated that it was not worthwhile to pay more just to see her/him every time because next time they will most probably meet the same one. On the other hand, those who have “rare” do not probably feel the advantage of meeting the same doctor every time. On the other hand, those who have “Never” might feel they need the same doctor most and most value meeting the same doctor every time.

The negative sign of the coefficients of the DPRSC and the Chance of Recovery scores were expected. Patients were willing to pay less than 0.002 for DPRSC (significant at the 5% level) and 0.007 for Chance of Recovery (significant at the 1% level). Moreover, the patients expressed negative willing to pay −6.2% for DPRSC (significant at the 5% level) and −1.67 for Chance of Recovery (significant at the 1% level). This means that the probability that a patient declares a positive WTP value decreases as the DPR-score or the Chance of Recovery score increase – a higher DPR - and Chance of Recovery-scores indicate a better satisfaction from the relationship with the doctor and a higher expected chance of recovery, respectively.

Finally, in marginal effects for drug availability, results suggest that the probability that patients were willing to pay in order to be “none” and “some” were respectively 21% and 6%. Drug availability for “none” was significant at the 1% level. Moreover, drug availability for “none” and “some”, patients were willing to pay 56.03 and 19.84 BDT respectively; where the former was significant at the 1% level.

Females had a tendency to state lower WTP values for improvements over the Attitude of the staff (−0.2%), seeing the same health professional (−2%), Doctor-Patient Relationship (−3%) and Chance of Recovery (−8%) (significant at the 5% level) attributes and state higher WTP values for improvements over the Geographical Proximity (2%), Waiting Time (4%), and Drug Availability (4%). Females expressed negative willing to pay for the Staff Attitude (−0.10 BDT), see the same health professional (−1.44 BDT), Doctor-Patient Relationship (−8.39 BDT) and Chance of Recovery (−17.64 BDT; significant at 5% level). Females also expressed positive willingness to pay for Geographical Proximity (1.17 BDT), Waiting Time (2.52 BDT), and Drug Availability (13.11 BDT). However, the sex variable was not significant.

In general, elderly patients had a lower probability of stating negative WTP values for improvements over all of the attributes except Drug Availability (0.01%). Other attribute improvements are: geographical proximity (−0.3%) (significant at the 1% level), waiting time (−0.2%) (significant at the 10% level), staff attitude (−0.2%), seeing the same health professional (−0.3%) (significant at the 5% level), doctor-patient relationship (−0.1%) (significant at the 10% level) and chance of recovery (−0.06%). Moreover, elderly patients expressed a positive willing to pay for Drug Availability of 0.03 BDT and expressed a negative willing to pay for geographical proximity of −0.21 BDT (significant at the 1% level), waiting time −0.12 BDT (significant at the 10% level), staff attitude −0.08 BDT, seeing the same health professional −0.16 BDT (significant at the 5% level), doctor-patient relationship −0.34 BDT (significant at the 10% level) and chance of recovery −0.13 BDT.

Higher educated patients were willing to pay more than lower educated patients to benefit from improvements over: geographical proximity (5%), waiting time (1%) (significant at the 10% level), staff attitude (0.09%) and seeing the same health professional (0.3%) attributes. On the other hand, higher educated patients were willing to pay less to benefit from improvements in the doctor-patient relationship (−0.4%), drug availability (−0.2%) and chance of recovery (−0.1%) attributes. Moreover, higher educated patients expressed a positive willing to pay for geographical proximity (0.31 BDT), waiting time (0.54 BDT), staff attitude (0.03 BDT) and seeing the same health professional (0.16 BDT). Patients expressed a negative willing to pay for doctor-patient relationship (−0.90 BDT), drug availability (−0.81 BDT) and chance of recovery (−0.22 BDT).

The income variable had a positive coefficient in all seven Tobit regressions. This was expected. However, the income variable was not very significant. Patients living in rural areas declared higher WTP values for lower waiting times (1%) and being able to meet the same health professional (1%). On the other hand, they were willing to pay less than the patients living in urban areas to benefit from improvements over all other attributes: geographical proximity (−6%), staff attitude (−40%), doctor-patient relationship (−5%), drug availability (−5%) and chance of recovery (−3%). However, the location variable was not significant. Moreover, patients living in rural areas expressed a positive willing to pay for waiting time (0.96 BDT) and same health professional (0.90 BDT). Patients living in rural areas expressed a negative willing to pay for geographical proximity (−4.04 BDT), staff attitude (−1.61 BDT), doctor-patient relationship (−12.78 BDT), drug availability (−16.47 BDT) and chance of recovery (−8.34 BDT).

Patients receiving health care from private hospitals were willing to pay more to improve the doctor patient relationship (7%) (significant at the 5% level) and chance of recovery attributes (5%), and less for geographical proximity (−2%), waiting time (−13%) (significant at the 5% level), staff attitude (−13%) (significant at the 5% level), seeing the same health professional (−13%) (significant at the 5% level), and drug availability (−3%) attributes, compared to those attending governmental facilities. Moreover, the private hospital patients expressed a positive willing to pay for the doctor patient relationship (17.77 BDT) (significant at the 5% level) and chance of recovery attributes (11.94 BDT). Patients receiving health care from private hospitals expressed a positive willing to pay for geographical proximity (−1.18 BDT), waiting time (−7.24 BDT) (significant at the 5% level), staff attitude (−5.23 BDT) (significant at the 5% level), seeing the same health professional (−6.80 BDT) (significant at the 5% level), and drug availability (−10.21 BDT).

Finally, the probability that patients coming to the hospital for an acute or common illness were willing to pay less than those who come to the hospital due to other reasons to benefit from improvements in: geographical proximity (−5%), seeing the same health professional (−4%), doctor-patient relationship (−3%), drug availability (−3%) and chance of recovery (−2%) attributes. On the other hand, acute patients were willing to pay more than others to benefit from improvements over: waiting time (0.5%), and staff attitude (3%) attributes. Moreover, acute patients expressed a positive willing to pay for waiting time (0.27 BDT) and staff attitude (1.54 BDT). Acute patients expressed a positive willing to pay for geographical proximity (−3.34 BDT), seeing the same health professional (−2.11 BDT), doctor-patient relationship (−6.75 BDT), drug availability (−9.27 BDT) and chance of recovery (−4.90 BDT).

## Conclusions

This study provides important information about the monetary valuation of seven quality attributes of health services by Bangladeshi health consumers. One of the assumptions in this study is the inter-attribute independence, i.e. the value of improvements over one attribute does not depend on the level of other attributes. However, a patient might value improvements in attribute over another depending upon how well the service is appreciated compared to the other attribute/s. Further research is needed to verify the existence of such inter-attribute dependence. However, the practical implications of this paper will give readers an opportunity to observe real patients’ behaviours using different attributes separately and to compare their satisfaction between sectors (public/NGO run centres versus private sector).

User fees play a major role in health care in Bangladesh. Among seven quality attributes, consumers were willing to pay more to improve three quality attributes viz. doctor patient relationship, drug availability and chance of recovery. To assess doctor patient relationship (DPRSC) score and chance of recovery (RECOVSC) score patients’ were asked to state whether they “strongly disagree”, “disagree”, “undecided”, “agree” or “strongly agree” on five Likert questions and were coded as 1 to 5, respectively. The negative coefficient of the chance of recovery (−3.20, significant at the 1% level) indicates that patients declares a positive WTP value decreases when the chance of recovery score increases and a higher expected chance of recovery. This result also suggested that when the chance of recovery score decreased as assessed by the RECOVSC score patients willing to pay more to benefit from the doctor. The same interpretation is applicable for the doctor patient relationship (−1.05, significant at the 5% level). Patients who did not find any of their prescribed medications in the hospital were willing to pay more than those who found “some” or “all” of their medications. Females were willing to pay more than males for higher doctor patient relationship and chance of recovery, indicating a less elastic demand for women but the sex variable was significant only for the chance of recovery score at the 10% level. Similar females, older patients has less elastic demand for doctor patient relationship and chance of recovery score but was significant for the doctor patient relationship score at 10% level. Rural people were willing to pay more than urban people for those three attributes indicating a less elastic demand for rural but the location variable was not significant.

The results also indicate that more educated patients have a positive effect on those three attributes and patients with higher income levels are willing to pay more. Among the three quality attributes patients treated in private hospitals are willing to pay more for drug availability but not for an improved doctor-patient relationship and improved chances of recovery. These results indicate that patients treated in private hospitals were more or less satisfied with their current doctor patient relationship and chances of recovery. Patients seeking care for acute problems were willing to pay more than chronic patients for those three attributes although there were no significant differences between acute and chronic patients for the rest of the attributes.

The doctor patient relationship is critical for vulnerable patients as they valued this relationship to a large extent. However, social skills training for doctors is often neglected in the health curriculum in Bangladesh. Health policy in Bangladesh should consider the fiduciary relationship; i.e., doctors are expected and required to act their patient’s interest and relationships based on openness, trust and good communication would enable a stranger partnership between the client and service provider to occur.

In some cases, there is a lack of availability of essential drugs due to fluctuating production levels or prohibitive cost. Recently some major pharmaceuticals companies such as Beximco, Square, Incepta and Novartis have significantly increased medicine prices due to the high import price of raw materials and the appreciation of the dollar against Bangladeshi taka. For the 49% of Bangladeshi people living below the national poverty line, the effects of increasing medicine prices have been devastating. Across Bangladesh, a lack of drug price controls and monitoring in the selection of drugs by doctors have resulted in many patients not recovering appropriate treatment. In some cases generic drugs are freely available in some public medical facilities, but in some cases doctors prescribe expensive branded medicines, which patents have to buy. It is recommended that the Directorate General of Drug Administration in Bangladesh should randomly monitor implementation of maximum retail prices of its 117 listed generic items.

## Consent

Oral/written informed consent was obtained from the patient for the publication of this report.

## Additional files

Additional file 1:
**Quality attributes and their corresponding measurement scales.**
Additional file 2:
**The seven partial WTP valuation questions with hypothesis.**
