# Supplementary material for: Assessing willingness to pay for health care quality improvements
Source: BMC Health Serv Res. 2015 Feb 1;15:43. doi: 10.1186/s12913-015-0678-6 (PMC4332931; doi:10.1186/s12913-015-0678-6)
Supplement: Additional file 1: — Quality attributes and their corresponding measurement scales. [file 12913_2015_678_MOESM1_ESM.docx]

**Additional file 1**

**Quality attributes and their corresponding measurement scales**

| Attributes | Measurement Scale |
| --- | --- |
| 1. Geographical proximity | Very Far, Far, Average, Close, Very Close. |
| 2. Waiting time | Very Long, Long, Average, Not Long, Not Long at All. |
| 3. Attitude of hospital staff | Excellent, Good, Bad, Very Bad. |
| 4. Being able to see the same doctor | Always, Often, Rarely, Never. |
| 5. Being able to discuss her/his problem with the doctor and receive sufficient information about her/his health status and the prescribed treatment(s) | Multi-item Likert-scaling; continuous: range [20,100]. Items: 1. I stayed sufficient time with the doctor. 2. The doctor explained to me my health problem. 3. The doctor explained to me how to use the prescribed treatment(s). 4. The doctor explained to me what I should do to prevent (or not to complicate) my health problem in the future. 5. The information was clear and sufficient. |
| 6. Being able to purchase the prescribed treatment(s) at the hospital within the range of prescribed fee | All, Some of Them, None. |
| 7. Chance of Recovery | Multi-item Likert-scaling; continuous: range [20,100]. Items: 1. I usually recover after being examined by the doctor of the hospital. 2. Many times, I need to go to a private clinic to be re-examined by a better doctor. 3. The doctor who examined me was a good doctor who knows what he is doing. 4. Private doctors are more competent. 5. In general, I prefer to go to private clinic. |
