# Supplementary material for: Assessing willingness to pay for health care quality improvements
Source: BMC Health Serv Res. 2015 Feb 1;15:43. doi: 10.1186/s12913-015-0678-6 (PMC4332931; doi:10.1186/s12913-015-0678-6)
Supplement: Additional file 2: — The seven partial WTP valuation questions with hypothesis. [file 12913_2015_678_MOESM2_ESM.docx]

**Additional file 2**

**The seven partial WTP valuation questions with hypothesis**

| 1. Benefit from a hospital similar to this one and located “Very Close” to your home? 2. Have a hospital with a “Waiting Time that you estimate as “Not long at All”? 3. Benefit from an “Excellent” attitude from the hospital staff? 4. Be able to see the same health professional every time you come to the hospital? 5. Be able to stay sufficient time with the doctor to discuss with him your health problem, receive sufficient and clear information about your disease and the prescribed treatment(s)? 6. Be able to find the prescribed treatment(s) “Always” available in the hospital within the range of prescribed fee? 7. Be examined by a more competent doctor and to have a higher chance of recovery? | “Yes” → “No” WTP = 0 | What is the maximum amount of money that you would be willing to pay (knowing that this extra amount of money will be paid at every coming visit), extra to what you currently pay, in order to… |
| --- | --- | --- |
